# Microneurovascular reimplantation in a case of total penile amputation

**DOI:** 10.4103/0970-0358.44945

**Published:** 2008

**Authors:** Yogesh C. Bhatt, Kinnari A. Vyas, Rajat K. Srivastava, Nikhil S. Panse

**Affiliations:** Department of Plastic Surgery, SSG Hospital and Medical College, Baroda, India

**Keywords:** Microneurovascular surgery, penile-replantation, total amputation

## Abstract

Amputation of the penis is a rare condition reported from various parts of the world as isolated cases or small series of patients; the common aetiology is self-mutilating sharp amputation or an avulsion or crush injury in an industrial accident. A complete reconstruction of all penile structures should be attempted in one stage which provides the best chance for full rehabilitation of the patient. We report here a single case of total amputation of the penis, which was successfully reattached by using a microsurgical technique. After surgery, near-normal appearance and function including a good urine flow and absence of urethral stricture, capabilities of erection and near normal sensitivity were observed.

## INTRODUCTION

Total penile amputation is an uncommon injury;[1–6] 87% of the patients reported had psychiatric problems. Self-amputation of external genitals is also known as Klingsor syndrome.[4–7] A few patients had poor gender identity feeling themselves inadequate as males. Some cases arise from felonious assault by jealous homosexual lovers.[1,6] In 1970 in Thailand, an epidemic was seen, of penile amputation as punishment for philandering by humiliated wives.[2–6] Microvascular penile replantation offers the best prospect for restoration of micturition function, return of sensations and erectile functions.

## CASE REPORT

**Figure 1:**
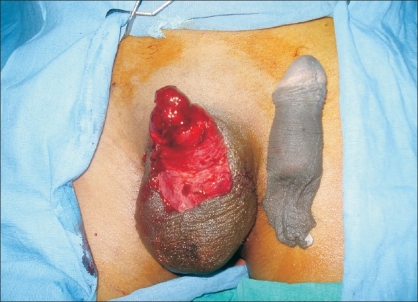
Total Amputation Of Penis

We report here a single case of total amputation of the penis [Figure 1] that was successfully reattached by using the microsurgical technique. In March 2007, a 22 year-old married male patient having three children presented to the casualty department with total amputation of his penis following assault. The amputated part was transported in a clean plastic bag immersed in ice. The scrotum with its testicles was found to be intact. Bleeding from the penile stump was stopped with the help of a pressure dressing and replantation of the amputated penis was attempted.

### Surgical technique

**Figure 2:**
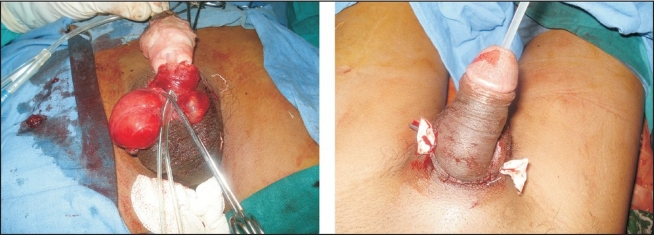
Urethral Repair

**Figure 3:**
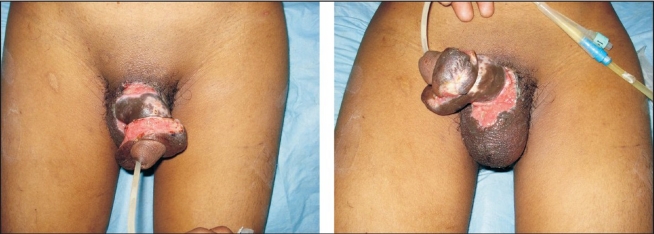
Post Release Incision Raw Areas

**Figure 4:**
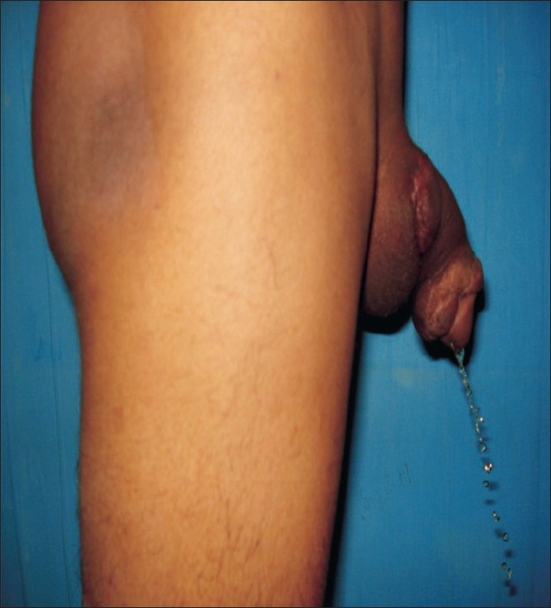
Micturition at 1 year

**Figure 5:**
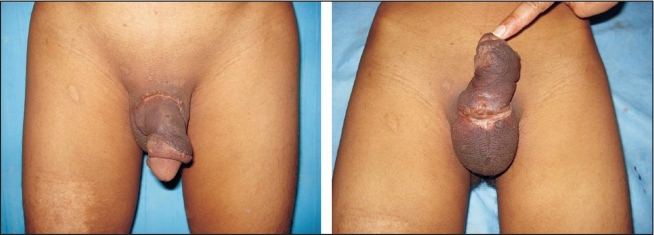
Cosmetic Appearance at 1 year

**Figure 6:**
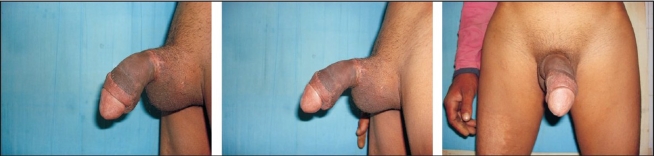
Penile Erection

The proximal penile stump and the amputated penis were assessed under the microscope, and the superficial and deep dorsal veins and both dorsolateral neurovascular pedicles were tagged. A no.14 silicone catheter was inserted transurethrally through the distal amputated part and urethral repair was done with 5-0 vicryl with inverting sutures [Figure 2]. The repair of corporal bodies was done circumferentially with 3-0 vicryl. In our case, replantation was accomplished by sequential end-to-end anastomosis of the deep dorsal vein, followed by the two dorsal arteries and finally the superficial dorsal vein with 9-0 Ethilon^®^. Microneural co-aptation of both the dorsal nerves of the penis was done with 9-0 Ethilon^®^. A drain was kept and loosely approximating skin sutures taken with Ethilon^®^ 3-0 to complete the replantation [Figure 2]. Loose dressings with supports kept the penis elevated; the whole procedure lasted four hours. Postoperative adjunctive measures were adequate hydration, administration of low molecular weight dextran at the rate of 20 mL/h for five days. Hourly monitoring was done by assessing colour, temperature and bleeding on pin prick when needed. Oedema over the glans and shaft of penis was observed on postoperative day 1, for which bilateral release incisions were given deep to the bucks fascia on both sides at the junction of the shaft and the glans, creating raw areas [Figure 3]. On the seventeenth postoperative day, the raw areas were debrided and primary suturing at the dorsal and ventral aspects of the shaft of the penis was done. Residual raw areas were covered with thick split thickness skin graft. Foleys catheter was removed after four weeks with good urine flow from the urinary meatus. On follow-up after one year, the urine flow [Figure 4] and cosmetic appearance [Figure 5] was good. Penile sensations showed recovery with appreciation of fine touch. The patient reported the restoration of his penile erection [Figure 6] and ejaculation during sexual intercourse.

## RESULT

The outcome of microsurgical repair was adequate cosmetic restoration of penis with good patient acceptability and micturition function. The recovery of penile sensations was good; suboptimal penile turgor was present at erection.

## DISCUSSION

**Figure 7:**
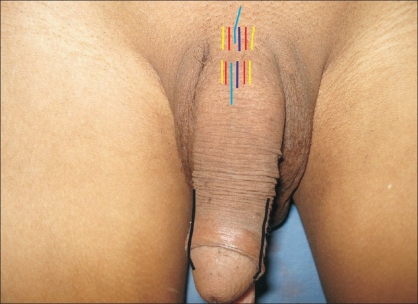
Line diagram showing neurovascular repair, release incisions

**Figure 8:**
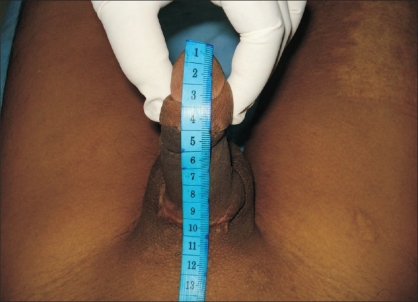
Total penile length

**Figure 9:**
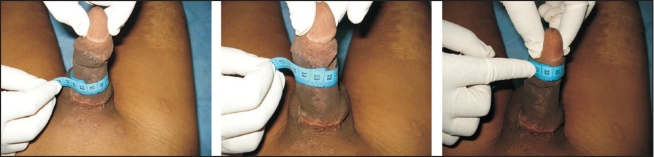
Penile girth

The first documented case of macroscopic penile replantation was reported in 1929 by Ehrlich.[4] Cohen *et al,* reported the first microvascular replantation of penis in 1977.[8] A review of the literature revealed that 80 cases underwent penile replantation, of which 30 cases underwent microsurgical replantation since 1970. These 30 cases have been reported to be of higher quality in terms of both functional and aesthetic result.[4,5] Many factors contribute to favourable final outcomes.[9] Analysis of our case revealed that the cleanly incised injury with a short duration of cold ischemia was an important factor that influenced the outcome. Another factor was the concept of microsurgical reapproximation. The macrosurgical replantation of the penis depends on corporal sinusoidal blood flow with the distal amputated part as a composite graft leading to high complication rates of skin necrosis, fistula formation, loss of sensations and erectile dysfunction. In contrast, the microsurgical approximation of the penile shaft structures provides early restoration of blood flow with the best prospects for graft survival, normal erectile function and optimal benefits with fewer complications.[4] Another critical factor for the success of replantation was the adequacy of venous outflow and the sequence of microsurgical anastomosis. Due to the dual vascular drainage in the penis, the superficial and deep dorsal veins, tributaries of saphenous and santorini plexus respectively, were both anastomosed for good venous return [Figure 7]. We suggest doing anastomosis of the dorsal veins before the dorsal penile arteries to prevent stasis.[4] The return of penile sensations over the glans was as expected in the yearly follow-up of our case with a distal amputated length of approximately 10 cm of total penile length [Figure 8]. In our opinion, another important factor was the critical postoperative monitoring of the replantation. Timely intervention was done in the form of release incisions to relieve oedema and maintain vascularity of the penis [Figure 7]. The initial raw areas may appear as disfiguring but the final result was satisfactory, with near uniform girth of the penile shaft [Figure 9]. We suggest similar measures to protect the anastomosis and prevent failure. Prophylactic release incisions can be an option when regular monitoring is not contemplated.

The adverse effect seen in our case was the skin loss due to necrosis of the proximal part of the penile skin, probably because we had anastomosed only the deep dorsal arteries, which are branches of the internal iliac artery. The external pudendal vessels were not anastomosed. It may be advisable to anastomose the superficial system also to avoid skin necrosis. The microsurgical restoration of penile vascularity provides early restoration of blood flow with the best prospects for graft survival, normal erectile function and optimal benefits due to fewer complications.

## CONCLUSION

The current concept of microvascular replantation for penile amputation is the treatment of choice with the best prospects for cosmetic restoration, physiological micturition and preservation of sensation and erectile function.
